# Non-IgE/Mixed Food Allergies and Functional Gastrointestinal Disorder: A Common Thread between Childhood and Adulthood

**DOI:** 10.3390/nu14040835

**Published:** 2022-02-16

**Authors:** Giacomo Caio

**Affiliations:** 1 Department of Translational Medicine, University of Ferrara, 44124 Ferrara, Italy; caigmp@unife.it; 2 Mucosal Immunology and Biology Research Center, Massachusetts General Hospital-Harvard Medical School, Boston, MA 02114, USA

Adverse food reactions (AFRs) are abnormal clinical responses related to food or the ingestion of a food component, including drinks, food additives, and dietary supplements [1,2]. AFRs can be classified as intolerances (non-immuno-mediated) or allergies (immune-mediated) depending on the pathophysiological mechanism of action [1,2]. Food intolerances are adverse responses to a food component in which the human immune system does not play a direct role, are often dose-dependent, and are not consistently reproducible. They can be caused by several mechanisms, such as the peculiar properties of an ingested food (i.e., toxic or pharmacologic active component, such as caffeine or alcohol), the lack of a particular enzyme with which to digest nutrients, or nutrients being too abundant to be completely digested [1,2]. Nonetheless, it is time to recognize gut microbiota changes (i.e., dysbiosis) as one of the major mechanisms contributing to the development of food intolerances and a key event in shifting a potential food intolerance (e.g., genetically lacking lactase) from a subclinical/asymptomatic form into an overt symptomatic condition. In this line, it is well-known that gut microbiota plays a paramount role in human metabolism and digestion by contributing enzymes that are not encoded by the human genome (e.g., enzymes for the breakdown of polysaccharides, oligo-di-mono-saccharides, polyols, FODMAPs, and other nutrients) [3].

On the other hand, a food allergy (FA) is a reproducible adverse health effect arising from a specific immune system response. Based on the immunological mechanism involved, an FA may be classified as: (a) IgE-mediated, which is one that is mediated by antibodies belonging to immunoglobulin E (IgE); (b) non-IgE, a heterogeneous group of food allergies in which there is an immune reaction against food components, but where the primary pathogenesis is not a product of IgE and thought to act mainly through cell-mediated mechanisms; and (c) mixed, in which both IgE-mediated and cell-mediated immunological mechanisms are involved in the reaction [1,2,4].

Non-IgE-mediated and mixed FAs are still a tough challenge for clinicians. The diagnosis of non-IgE and mixed FAs remains one of exclusion and is mainly clinical, except for food-protein-induced enteropathy (FPE) and eosinophilic gastrointestinal disorder (EGID), in which histological confirmation is required. Unfortunately, non-IgE and mixed FAs are frequently implicated in causing gastrointestinal symptoms in children and adults but remain likely underdiagnosed, and evidence-based protocols with which to diagnose and treat these diseases are lacking [5].

The clinical presentation of non-IgE and mixed FAs is characterized by significant gastrointestinal and extraintestinal symptoms that disappear after starting an elimination diet (most frequently the withdrawal of cow’s milk, egg, soy, wheat, and corn) and reappear when the culprit allergen is reintroduced [5]. In this line a double-blind placebo-controlled food challenge (DBPCFC) is still considered the diagnostic gold standard, not only for non-IgE and mixed but even for well-known IgE-mediated FAs. [1,2].

Looking at pediatric practices, an interesting review published by Calvani et al. in *Nutrients* [6] thoroughly describes gastrointestinal non-IgE-mediated and mixed FAs in infancy and early childhood. They provide an updated and detailed overview on the different clinical pictures, possible diagnostic tools, and pediatric clinical scores (with recognized strict limitations and uncertainties) of non-IgE and mixed gastrointestinal FAs in the first years of life, embracing food-protein-induced allergic proctocolitis (FPIAP), food-protein-induced enteropathy (FPE), food-protein-induced enterocolitis syndrome (FPIES), and eosinophilic gastrointestinal disorders (EGID), such as eosinophilic esophagitis (EoE), eosinophilic gastroenteritis (EGE), and eosinophilic colitis (EC).

The clinical scenario of non-IgE and mixed allergies in both the pediatric and particularly the adult population is not limited to the six thoroughly described diseases presented in the paper by Calvani [6], which are just the tip of the iceberg. Clinicians know that in clinical practice several patients ask for help, struggling with both intestinal and extraintestinal symptomatology claimed to be related to some food hypersensitivity. For decades these kinds of patients, characterized by the absence of “classic” IgE-mediated mechanisms and normal endoscopic findings, were relegated to the broad area of functional gastrointestinal disorders (FGIDs) (e.g., irritable bowel syndrome (IBS), functional dyspepsia (FD), etc.) [7,8].

In recent years, recent advances in FGIDs have demonstrated micro-organic alterations at the biopsy level with different characteristics from organic intestinal diseases (e.g., celiac disease, Crohn’s, etc.), which pathologists often do not recognize in a routine examination, thus labeling them as “normal mucosa” or “mucosa with non-specific chronic inflammatory infiltrate”. An increase in eosinophil and mast cell count was demonstrated in a subgroup of patients with FGIDs along with local and systemic cytokine changes [9,10,11,12,13]. Moreover, cellular immune activation with increased small bowel homing T cells has been demonstrated to be possible key factors in the clinical manifestations of IBS and FD [14]. Intestinal barrier impairment and microbiota changes (i.e., leaky gut—dysbiosis syndrome) have been documented in patients with FGIDs, and this could allow the penetration of microbial and food allergens into the mucosa, triggering a local inflammatory response that in some cases could spread at the systemic level, predisposing one to a loss of tolerance for self- (autoimmunity) and non-self-innocuous antigens (allergy) [15]. Intriguingly, food antigens/allergens might induce acute changes in intestinal permeability, as demonstrated using a confocal laser endomicroscopy (CLE) analysis in a population labeled as affected by IBS [16]. More than 50% of these “IBS patients” could suffer from a sort of “non-IgE-mediated food allergy”, with the immediate disruption of the intestinal barrier upon exposure to food antigens (wheat, milk, and soy). An increased activation and degranulation of eosinophil also characterized the same cohort of patients, thus confirming a non-IgE-mediated allergic mechanism [16]. Interestingly, most IBS patients challenged with food during CLE analysis reacted to wheat, thus objectively confirming the existence of non-celiac wheat sensitivity (NCWS) [16,17,18,19,20]. In this line, some food hypersensitivities, such as NCWS, have clearly demonstrated the immune system’s involvement and, in this line, they should be classified as part of the vast bag of non-IgE allergies rather than as FGIDs [21,22]. Last but not least, a link between allergic disease in infancy and the future development of FGIDs in late childhood–adolescence has been recently established, implicating a possible shared pathophysiology among these disorders and possibly a sort of “new allergy march” parallel to the well-known “classic atopic march” [23,24].

These insights fuel a contagious enthusiasm in the scientific community. Eliminating some key food allergens, demonstrated to be involved in acute microorganic changes in intestinal barrier function via CLE analysis, could be a way to manage patients more effectively in the future. Researchers should approach this intriguing topic by leaving behind the confines of classic allergology and gastroenterology “dogmas” and preconceptions, and a joint effort involving allergists, gastroenterologists, and immunologists on these topics is needed.

## Data Availability

Not applicable.
